# Quick-Delivery Mold Fabricated via Stereolithography to Enhance Manufacturing Efficiency

**DOI:** 10.3390/mi15111345

**Published:** 2024-10-31

**Authors:** Jae Hyang Lee, Seonghwan Park, Jeon Taik Lim, Hyung Sun Yoon, Jae Won Choi, Cheol Woo Ha, Jiyong Park

**Affiliations:** 1 Advanced Joining and Additive Manufacturing R&D Department, Korea Institute of Industrial Technology, 156 Gaetbeol-ro, Yeonsu-gu, Incheon 21999, Republic of Korea; 2 Department of Energy Engineering, Hanyang University, Seoul 04763, Republic of Korea; 3 Department of Convergence Manufacturing System Engineering, University of Science and Technology (UST), 217 Gajeong-ro, Daejeon 34113, Republic of Korea; 4 Lincsolution, 41-4, Burim-ro 170beon-gil, Dongan-gu, Anyang-si 14055, Republic of Korea

**Keywords:** stereolithography (SLA) mold, post-thermal curing process, degree of cure, injection molding test

## Abstract

The ever-growing demand for reducing costs and decreasing the time to market in today’s plastics industry makes rapid tooling and rapid prototyping highly researched areas. Stereolithography (SLA)-manufactured injection mold inserts make it possible to produce prototype parts rapidly and cost-effectively. To utilize SLA in the injection molding industry, two steps have to be considered. The first is to identify suitable SLA process and post-thermal curing process parameters to enhance the mechanical and thermal characteristics. The second is to verify the applicability of SLA-manufactured molds for use in the injection molding industry. IA comprehensive study was performed to find the optimum process parameters for an SLA mold with excellent mechanical and thermal properties and to verify the applicability of the mold. First of all, the mechanical and thermal properties of samples manufactured based on various laser powers and heat treatment at different temperatures were analyzed with a tensile test, DSC, and TMA according to the degree of cure. On the basis of the results from those tests, an SLA mold was designed and fabricated with the optimum mechanical and thermal properties. In addition, the SLA mold was assembled into an injection machine, and an injection molding test was conducted. The SLA mold endured during the injection cycle, and 500 shots were successfully injected without damaging the mold, which resulted in reaching the quick-delivery mold standard. Finally, we demonstrate that SLA is an effective technology to produce molds for use in the injection molding industry.

## 1. Introduction

The shortened lifespan of products and the need for quicker product development in injection molding have heightened the appeal of rapid tooling [1]. By combining rapid prototype tooling with injection molding, the costs and lead times traditionally associated with conventional tool manufacturing techniques like machining or electro-discharge machining are significantly decreased. This integration can cut down tool delivery time by at least 50 percent [1]. Therefore, rapid tooling technologies are of great importance for injection molding. In this context, additive manufacturing (AM) technologies have been gaining much attention because of their several benefits compared with those of traditional manufacturing for mold and insert fabrication, including waste reduction and reduced overall manufacturing time and costs [2]. Among the AM technologies, stereolithography (SLA), which works by solidifying a liquid resin selectively using UV light exposure, is particularly advantageous for creating large, intricate, high-precision parts with superior surface quality at a reasonable cost and without losing processing resolution, so SLA would be a good choice to fabricate molds [2,3,4,5]. In this AM process, soft materials, including a polymer, are used, but they present thermal and mechanical properties that are substantially different from those of metals, influencing the mold’s durability at elevated temperatures and the quality of the final products, such as geometrical dimensions [6]. As injection molding molds endure specific process conditions involving a flow pressure with high thermal stress [7], it is of great importance to investigate the mechanical and thermal properties of SLA-manufactured molds and to verify the applicability of the molds for use in the injection molding industry.

In SLA-fabricated parts, the degree of cure should be considered to obtain good mechanical and thermal properties because these characteristics are highly dependent on the degree of cure [8]. However, even though green parts (after being built via SLA) are fabricated based on optimum SLA parameters, these materials are not fully cured, leading to a decline in the mechanical and thermal properties. Also, if a mold that is not fully cured is used during the injection molding process, the mold can be subject to shrinkage, which may change its dimensions and affect the overall accuracy of the products. Thus, a post-cure procedure is necessary to enhance the mechanical and thermal characteristics and to ameliorate the accuracy of the products. Some previous researchers have examined the relationship between the degree of cure after different post-cure procedures and mechanical and thermal properties. Riccio C. et al. [8] investigated the effect of different curing processes on the mechanical properties of SLA resins. Miedzińska D. et al. [9] also evaluated the influence of curing time on the mechanical behavior of SLA-fabricated samples at different strain rates. Also, according to a study [10], the SLA curing process is incomplete. Thus, post-cure processes are necessary to ensure the complete cure of samples and increase the material’s properties. In this previous study, two types of post-curing processes (without any source of light and exposure to UV light) were conducted, proving effective in making the mechanical properties better. Furthermore, Zirak Nader et al. [11] investigated the thermal and mechanical properties of cured (meth)acrylate-based photocurable resin with the use of SLA. It was found that the post-curing process increased the mechanical and thermal properties.

In trying to verify the applicability of molds manufactured via SLA, the injection molding test should be carried out. There exist some studies aimed at the evaluation of rapid-prototyped injection molds fabricated with SLA and PolyJet technologies. Surace R. et al. [2] performed micro-injection molding tests for the evaluation of an SLA-manufactured mold. Moreover, Gheisari R. et al. [12] investigated stereolithography tools for micro-injection molding, and the number of shots before mold failure was five. Kampker, Achim et al. [13] focused on a comparison between a part’s materials properties from conventionally milled aluminum tools and polymer inserts manufactured via PolyJet technology. Krizsma Sz et al. [14] carried out a novel test series on PolyJet-fabricated injection mold inserts to examine the effects of thermal expansion on mold insert deformation in real time. Mendible et al. [1] investigated the thermal behavior of inserts manufactured via rapid tooling techniques, such as PolyJet and direct metal laser sintering (DMLS). In addition, in previous studies [15] a micro-injection molding test was performed, involving the injection molding of polyethylene (PE) under 100 shots. However, it is difficult to find previous research on large-scale injection molding tests. Thus, comprehensive research is required, involving investigating the SLA process and post-thermal curing parameters for manufacturing SLA molds with optimum mechanical and thermal properties and verifying the applicability of SLA molds in the injection mold industry on a large scale.

A comprehensive study was conducted to find the proper process parameters for an SLA mold with excellent mechanical and thermal properties and to verify the applicability of the mold on a large scale. This work, described in the present paper, was split into two sections. It began with an investigation of the mechanical and thermal properties of samples manufactured via SLA under various SLA process parameters and post-thermal curing conditions. And, an injection molding test of the SLA mold was performed to prove the potential to make use of SLA technology in the injection molding industry on a large scale.

## 2. Experimental Section

### 2.1. Sample Preparation

**Figure 1:**
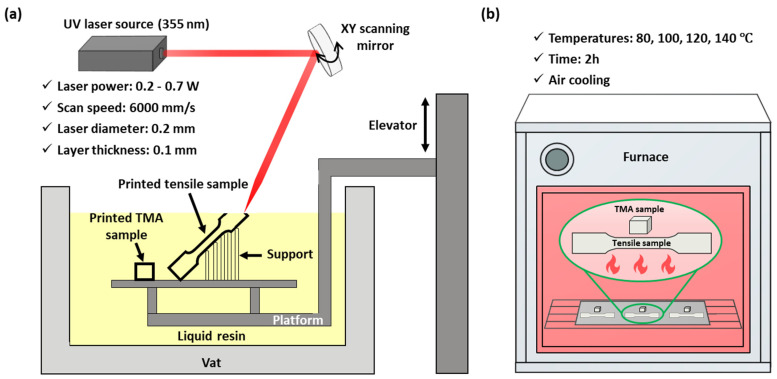
Schematic illustrations of (**a**) SLA and (**b**) heat treatment processes.

A photoresin, Formula L1 (ABS-like) (PROTOFAB, Hwaseong, Republic of Korea), was 3D-printed by an SLA 3D printer (LINK SL-1500, LINCSOLUTION, Anyang, Republic of Korea) to produce specimens for tensile, differential scanning calorimeter (DSC), and thermomechanical analyzer (TMA) tests. In this present study, all samples were manufactured at different laser power levels: 0.2, 0.3, 0.4, 0.5, 0.6, or 0.7 W, with all other process parameters held constant. Other process conditions included a laser scan speed of 6000 mm/s, a laser diameter of 0.2 mm, a layer thickness of 0.1 mm, and an inclination of 60 ° to the printing plane. These samples are hereafter referred to as 0.2 W, 0.3 W, 0.4 W, 0.5 W, 0.6 W, and 0.7 W, respectively. The tensile samples were designed in a computer-aided design (CAD) system and manufactured according to ASTM D638 [16]. Immediately after the SLA process, all samples were washed with 2-propanol. Then, 0.5 W was selected and heat-treated at various temperatures (80, 100, 120, or 140 °C) for 2 h because of the relatively better mechanical properties of the 0.5 W sample. The heat-treated samples were heat-treated 0.5 W at 80 °C, 100 °C, 120 °C, or 140 °C. Figure 1 presents an image of all samples before and after the heat treatment, showing the discoloration of the heat-treated 0.5 W samples at 120 °C and 140 °C.

### 2.2. Tensile Test

Tensile tests were conducted by a testing machine (Z010 TE, Zwick & Roell, Ulm, Germany) at room temperature and 5 mm/s crosshead speed. The elastic modulus was calculated by analyzing the relationship between stress and strain within the linear range. Strain was gauged using an incremental extensometer. The tensile tests for each sample were conducted three times to ensure data repeatability.

### 2.3. Differential Scanning Calorimeter (DSC) Analysis

DSC experiments were performed on a DSC Q200 (TA Instrument, New Castle, DE, USA) by heating three samples (0.5 W sample, and samples heat-treated at 0.5 W and at 100 °C and 120 °C) from 25 to 300 °C at a heating rate of 10 °C/min under a nitrogen flow (50 mL/min) in order to measure the degree of cure of those samples. The average specimen weight was around 5 mg. To make the DSC samples, each tensile sample was cut, and the middle position was taken. The percentage of curing in each sample was calculated from the following equation [17]:

(1) $$Deegree\;of\;cure\left(\%\right)=\left(1-\frac{∆H_{sample}}{∆H_{resin}}\right)\times 100$$

where $∆H_{sample}\;$ and $∆H_{resin}$ are the heat release energy from the samples and resin, respectively.

### 2.4. Thermomechanical Analyzer (TMA) Analysis

TMA experiments were conducted on a Q400 TMA instrument (TA Instrument, New Castle, DE, US) to evaluate the thermal stability by measuring the coefficient of thermal expansion (CTE), glass transition temperature (T_g_), irreversible thermal strain (ITS), and softening temperature (T_softening_) depending on the degree of cure of the three samples (0.5 W sample, 0.5 W samples heat-treated at 100 °C and 120 °C). The size of the cubic samples for the TMA tests was 5 mm×5 mm×3 mm. For the CTE and T_g_, those samples were heated from 30 to 180 °C at a heating rate of 10 °C/min and a 0.1 N static force. In addition, the ITS was calculated from the displacement values measured during the heating/cooling cycle [18]:

(2) $$ITS(\%)=\left(\frac{∆L}{L_{0}}\right)\times 100$$

where ∆L and $L_{0}$ are the change in length and initial length. And, two thermal process conditions for obtaining the ITS were applied in this present study. The first was the first heating/cooling cycle from 30 to 70 °C at a heating rate of 10 °C/min. The second was the isothermal heating process at 70 °C for 30 min at a heating rate of 10 °C/min, followed by the cooling. In an attempt to measure the T_softening_, a TMA penetration test was carried out. The penetration depth of a ball-point probe into the sample under an applied load of 1 N was measured as a function of temperature ranging from 20 to 70 °C at a heating rate of 2 °C/min. At the beginning of heating, the TMA curve was close to linear owing to the thermal expansion of the sample. Over time, the material softened, and the probe continued to penetrate the sample; then, at a point, the TMA curve declined rapidly. T_softening_ can be determined as the point of intersection of two tangent lines [18].

### 2.5. Injection Molding Test

**Figure 2:**
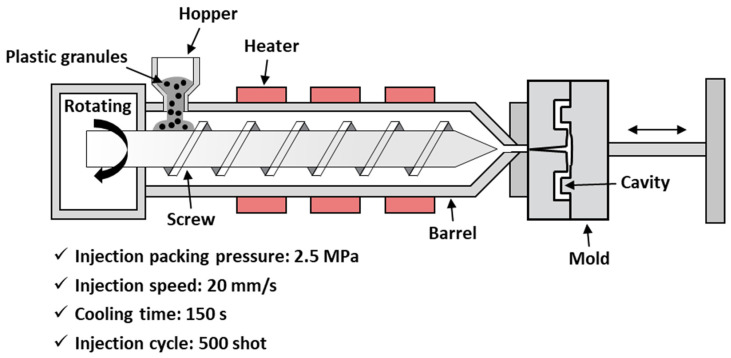
Schematic illustration of injection molding test.

To verify the feasibility of the SLA-manufactured mold, the mold set was equipped with an injection molding machine (TB200S, WOOJINPLAIMM Boeun, Republic of Korea). The injection packing pressure, injection speed, and cooling time were set to 2.5 MPa, 20 mm/s, and 150 s, respectively. The injection molding test was performed repeatedly until injection cycles reached 500 shots, which is standard for a quick-delivery mold (QDM), as shown in Figure 2.

## 3. Results and Discussion

### 3.1. Mechanical Properties Before and After Heat Treatment

**Figure 3:**
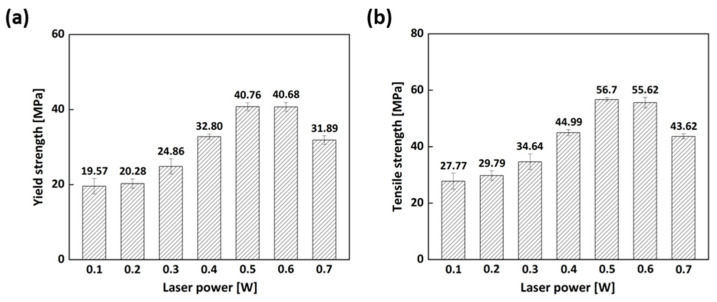
Mechanical properties of the samples fabricated with various laser powers before heat treatment: (**a**) yield strength; (**b**) tensile strength.

**Figure 4:**
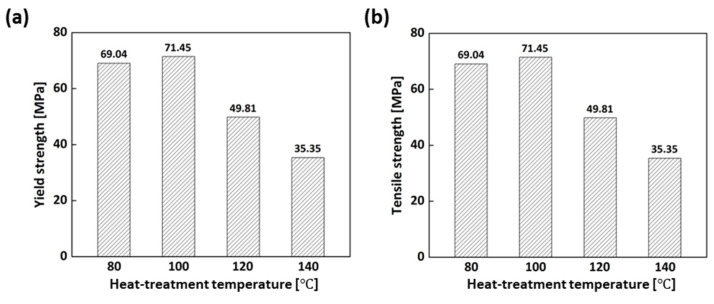
Mechanical properties of the heat-treated samples at various temperatures: (**a**) Yield strength; (**b**) Tensile strength.

For rapid tools such as injection molds, their mechanical properties, especially tensile strength, are of great importance to prevent premature failure during use. Figure 3 presents the mechanical properties of the samples manufactured with the various laser powers before heat treatment, showing the significant effect of laser power on the yield and tensile strength. The mechanical properties of the 0.5 W sample were higher than those of the others. When it comes to the 0.2 W, 0.3 W, and 0.4 W samples, poor mechanical properties were found because uncured resin between the layers remained in these samples, leading to a reduction in the mechanical properties [19]. In contrast, both strengths at a laser power of 0.7 W were found to decrease. The reason for the decline might be that increasing the laser power in SLA can generate higher local temperatures in the material during the curing process, result in faster polymerization and potentially greater shrinkage of the material as it solidifies, introducing higher levels of residual stress in the printed part, and resulting in the degradation of the mechanical properties. Considering the mechanical properties of the samples before the heat treatment, the laser power of 0.5 W was optimum, so the 0.5 W sample was selected and subjected to heat treatment at different temperatures in an attempt to increase the degree of cure and the mechanical properties. As can be seen in Figure 4, the yield strength of the heat-treated 0.5 W sample at 80 °C and 100 °C was increased by around 70% and 75%, respectively, compared with that of the 0.5 W sample. The tensile strength was also improved by approximately 22% and 26%, respectively, after the heat treatment. The improvement in the mechanical properties after the heat treatment could be attributed to increasing the degree of cure. However, when the samples were heat-treated at 120 °C and 140 °C, the properties declined. This could be ascribed to overheating. As a result, considering the mechanical properties of the heat-treated samples, the heat-treatment process at 100 °C for 2 h is optimum for obtaining higher yield and tensile strength.

### 3.2. DSC Analysis Before and After Heat Treatment

**Figure 5:**
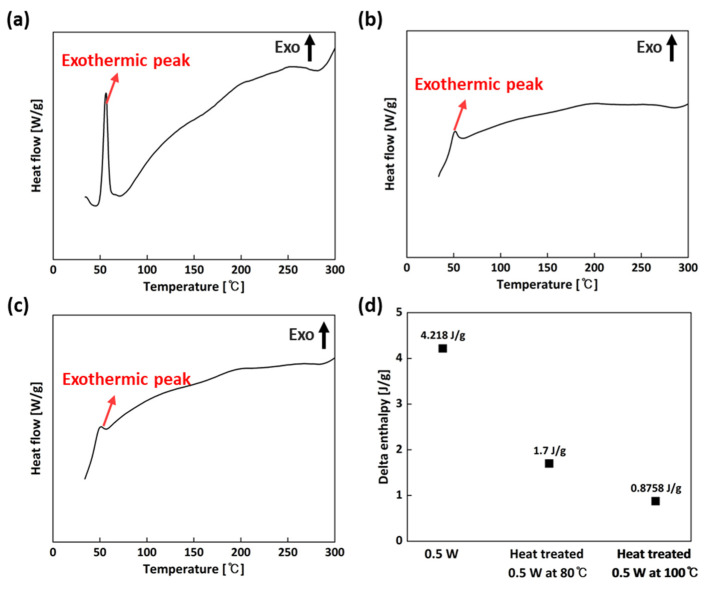
DSC curves for the 0.5 W sample and the samples heat-treated 0.5 W at 80 °C and 100 °C: (**a**) 0.5 W; (**b**) heat-treated 0.5 W at 80 °C and (**c**) at 100 °C; (**d**) delta enthalpy of the three samples.

Three samples (the 0.5 W, and the 0.5 W samples heat-treated at 80 °C and 100 °C) were selected, and their thermal properties were analyzed by DSC to measure the degree of cure that corresponded to the mechanical properties. It can be observed in Figure 5, the exothermic peak of the 0.5 W sample is relatively large compared to those of the heat-treated samples as a result of the energy released by the uncured resin regions in the 0.5 W sample. In other words, 0.5 W sample had a relatively high percentage of uncured resin, as indicated by a cure enthalpy of 4.218 J/g, in comparison with the heat-treated specimens. Also, as mentioned previously, the degree of cure could be calculated by Equation (1) ($∆H_{resin}$ = 47.65 J/g). As shown in Table 1, the sample heat-treated at 0.5 W and 100 °C had the highest degree of cure, in good agreement with the results from the mechanical tests. This is because increasing the heat-treatment temperature from 80 to 100 °C led to an increase in the polymerization during the heat treatment, which could be ascribed to increasing the mobility of the polymer chain by raising the heat-treatment temperature. Thus, the increase in the temperature increased the chain mobility, and, by raising the polymer network mobility, the chain transfer reaction was improved [11].

### 3.3. TMA Analysis Before and After Heat Treatment

**Figure 6:**
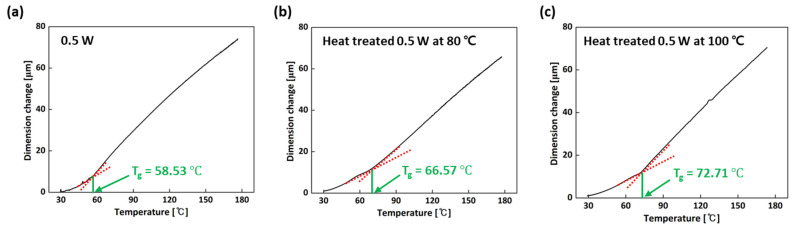
TMA curves for 0.5 W sample and samples heat-treated at 0.5 W and 80 °C or 100 °C: (**a**) 0.5 W; (**b**) heat-treated 0.5 W at 80 °C and (**c**) at 100 °C.

As injection molding molds withstand specific process conditions involving a flow pressure with high thermal stress, it is of great importance to evaluate the glass transition temperature (T_g_), irreversible thermal strain (ITS), softening temperature (T_softening_), and coefficient of thermal expansion (CTE) of samples in order to prevent premature failure and changing the mold’s dimensions, which can affect the overall accuracy of the products. With the use of TMA, the thermal stability can be evaluated by measuring the T_g_, ITS, T_softening_, and CTE. Figure 6 and Table 2 present the T_g_ and CTE values before the T_g_. When it comes to the T_g_, the temperature of a polymeric material can be assigned because the CTE showed a remarkable increase as the polymer proceeded through the temperature range related to the transition from the glassy to the rubbery state. The assignment of T_g_ involves the extrapolation of the slope of the TMA probe-displacement curve before and after the transition. It was found that the T_g_ of the sample heat-treated at 0.5 W and 100 °C (72.71 °C) was higher than that of the 0.5 W sample (58.53 °C) and the sample heat-treated at 0.5 W and 80 °C (66.57 °C) because the post-thermal curing process at 100 °C promoted an increase in the degree of cure. This densification of the polymer network might restrict molecular mobility, increasing the T_g_ of the material [8,20]. In addition, the CTE values before the T_g_ were calculated to be 100.1 ppm/℃ for the 0.5 W sample, 93.05 ppm/℃ for the sample heat-treated at 0.5 W and 80 °C, and 77.95 ppm/℃ for the sample treated at 100 °C, showing that the sample heat-treated at 0.5 W and 100 °C had the lowest CTE value. As the degree of cure increases, more polymer chains become cross-linked. Thus, the cross-linking restricts the mobility of the polymer chain in response to temperature changes. In other words, when the degree of cure raises, the polymer network becomes more rigid and less prone to expansion and contraction with temperature variations, leading to a decrease in the CTE. Therefore, the higher T_g_ and the lower CTE in the sample heat-treated at 0.5 W and 100 °C could be attributable to the higher degree of cure.

**Figure 7:**
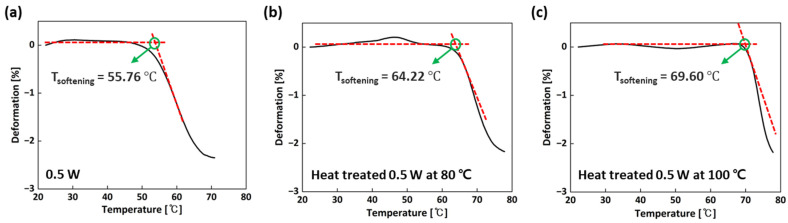
Penetration test curves for 0.5 W sample and samples heat-treated at 0.5 W and 80 °C or 100 °C: (**a**) 0.5 W; (**b**) heat-treated 0.5 W at 80 °C and (**c**) at 100 °C.

Also, through Equation (2), the ITS of the three materials could be calculated using TMA. There are two thermal process conditions used to measure the ITS values. The first is the first heating/cooling cycle from 30 to 70 °C at a heating rate of 10 °C/min. The second is the isothermal heating process at 70 °C for 30 min at a heating rate of 10 °C/min, followed by cooling. As shown in Table 3, the ITS values of the sample heat-treated at 0.5 W and 100 °C under the first heating/cooling cycle and the isothermal heating process were calculated to be 0.23% and 0.3%, respectively, which had the lowest values compared with those of the 0.5 W sample (0.83% and 1.06%). Because of the higher degree of cure in the sample heat-treated at 0.5 W and 100 °C, the CTE was lower, leading to fewer dimension changes and lowering the ITS value when this sample was exposed to the various thermal conditions. In addition, the T_softening_ of the three materials was obtained by a penetration test. The T_softening_ is related to the T_g_, which means the higher the T_g_, the higher the T_softening_. Figure 7 exhibits the penetration test curves and the T_softening_. It was found that the T_softening_ of the sample heat-treated at 0.5 W and 100 °C (69.60 °C) was higher than that of the other specimens, which could be attributed to the higher degree of cure. In summary, considering the mechanical and thermal characteristics, the heat-treatment process at 100 °C was most suitable for obtaining those higher properties and for manufacturing an SLA mold for injection molding tests.

### 3.4. Injection Molding Test of SLA Mold

**Figure 8:**
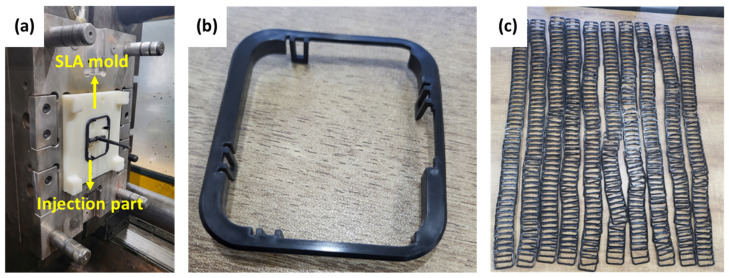
Photographs of the equipment of the SLA mold and injection part: (**a**) SLA mold; (**b**,**c**) Injection parts.

If an SLA mold is not suitable for injection molding, it is not worth fabricating the mold. So, to verify the applicability of the SLA mold, based on the mechanical and thermal property tests, the SLA mold was fabricated with the optimal process parameters (a laser power of 0.5 W and a post-thermal curing process at 100 °C for 2 h). Then, the injection molding test was carried out. As shown in Figure 8, the SLA mold endured the injection cycle, and 500 shots could be successfully injected without damaging the SLA mold. Therefore, the feasibility test results suggest the potential of the SLA mold for use in the injection molding industry.

## 4. Conclusions

A comprehensive study was carried out to find the optimal process parameters to manufacture an SLA mold with good mechanical and thermal properties and to verify the applicability of the mold. This study discussed here was divided into two parts. Firstly, the mechanical and thermal characteristics of samples produced through SLA using different process parameters and post-thermal curing conditions were investigated. Secondly, an injection molding test on the SLA was conducted to demonstrate the technology’s potential for the injection molding industry. The key conclusions are summarized below:

(1) Mechanical properties before and after the heat treatment:

When it comes to the yield and tensile strength of the samples before the heat treatment, the 0.5 W sample had the best mechanical properties. For the 0.2 W, 0.3 W, and 0.4 W samples, poor mechanical properties were found because of the uncured resin that degraded their mechanical properties. In contrast, the 0.7 W sample was also found to have decreased properties as a result of the residual stresses. And, after heat treatment at 80 and 100 °C for 2h, the properties increased compared with those of the 0.5 W sample. This might be attributed to the increased degree of cure after the heat treatment.

(2) Thermal properties before and after the heat treatment:

DSC was used to measure the degree of cure. It was found that the three samples (the 0.5 W sample, and the samples heat-treated at 0.5 W and 80 °C and 100 °C) exhibited an exothermic peak, but the exothermic peak of the 0.5 W sample was relatively large compared to those of the heat-treated samples because of the energy released by the uncured resin regions. Through the calculation of the delta enthalpy of the three samples, the degree of cure was measured, showing that the sample heat-treated at 0.5 W and 100 °C had the highest degree of cure due to the increase in polymerization during the heat treatment. Also, TMA experiments were performed to evaluate thermal stability by measuring the CTE, T_g_, ITS, and T_softening_. It was found that because of the higher degree of cure in the sample heat-treated at 0.5 W and 100 °C, the CTE and ITS were found to be lower, but the T_g_ and T_softening_ were calculated to be higher compared with those of the others. In summary, considering the mechanical and thermal properties, a laser power of 0.5 W and a post-thermal curing process at 100 °C were optimum to obtain those better properties and to manufacture an SLA mold for an injection molding test.

(3) Injection molding test for the SLA mold:

To verify the applicability of the SLA mold, an injection molding test was carried out. The SLA mold was produced based on the optimal process parameters (a laser power of 0.5 W and a post-thermal curing process at 100 °C for 2 h). As a result, the SLA mold endured the injection cycle, and 500 shots could be injected without damaging the SLA mold.

Therefore, the findings of this study suggest that SLA is an effective technology for use in the injection molding industry.

## Figures and Tables

**Table 1:** Degree of cure 0.5 W sample and 0.5 W samples heat-treated at 80 °C and 100 °C.

Specimen	Degree of Cure
0.5 W	91.14%
Heat-treated 0.5 W at 80 °C	96.43%
Heat-treated 0.5 W at 100 °C	98.16%

**Table 2:** T_g_ and CTE values of 0.5 W sample and samples heat-treated at 0.5 W and 80 °C or 100 °C.

Specimen	T_g_	CTE (30~T_g_ ℃)
0.5 W	58.53 °C	100.1 ppm/℃
Heat-treated 0.5 W at 80 °C	66.57 °C	93.05 ppm/℃
Heat-treated 0.5 W at 100 °C	72.71 °C	77.95 ppm/℃

**Table 3:** ITS values of 0.5 W, heat-treated 0.5 W at 80 °C and 100 °C.

Specimen	ITS (First Heating/Cooling Cycle)	ITS (Isothermal Heating Process)
0.5 W	0.83%	1.06%
Heat-treated 0.5 W at 80 °C	0.53%	0.62%
Heat-treated 0.5 W at 100 °C	0.23%	0.3%

## Data Availability

The original contributions presented in the study are included in the article, further inquiries can be directed to the corresponding authors.
